# Valve-in-valve procedures for degenerated surgical and transcatheter aortic valve bioprostheses using a latest-generation self-expanding intra-annular transcatheter heart valve

**DOI:** 10.3389/fcvm.2023.1209184

**Published:** 2023-09-01

**Authors:** Andreas Schaefer, Till Joscha Demal, Oliver D. Bhadra, David Grundmann, Lisa Voigtländer, Lara Waldschmidt, Johannes Schirmer, Simon Pecha, Yvonne Schneeberger, Niklas Schofer, Nils Sörensen, Stefan Blankenberg, Hermann Reichenspurner, Moritz Seiffert, Lenard Conradi

**Affiliations:** ^1^ Department of Cardiovascular Surgery, University Heart and Vascular Center Hamburg, Hamburg, Germany; ^2^ Department of Cardiology, University Heart and Vascular Center Hamburg, Hamburg, Germany

**Keywords:** transcatheter aortic valve implantation, bioprostheses, aortic valve, valve-in-valve, self-expandable aortic valve

## Abstract

**Background:**

Valve-in-valve (ViV) transfemoral transcatheter aortic valve implantation (TAVI) for failing aortic surgical bioprostheses or transcatheter heart valves (THV) has demonstrated a reasonable clinical and hemodynamic efficacy. Traditionally, self-expanding (SE) supra-annular THV are considered to result in superior hemodynamics compared with balloon-expandable intra-annular THV after ViV. However, so far no data are found on latest-generation intra-annular SE THV for aortic ViV procedures which might be superior with regard to coronary access or subsequent valve reintervention.

**Aim:**

We herein aim to evaluate a latest-generation SE intra-annular THV for aortic ViV procedures.

**Materials and methods:**

Between May 2022 and November 2022, five consecutive patients (4/5 female with mean age of 76.2 years and mean Society of Thoracic Surgeons predicted risk of mortality score of 2.9%) received ViV TAVI using the Navitor system (Abbott, Chicago, IL, USA) for treatment of failing surgical bioprostheses or THV. Data were retrospectively analyzed according to updated Valve Academic Research Consortium 3 (VARC-3) definitions.

**Results:**

At 30 days, absence of mortality and VARC-3 adjudicated clinical endpoints were documented. Echocardiography at 30 days revealed complete absence of paravalvular leakage and single-digit mean transvalvular gradients (mean of 6.0 mmHg) in all patients.

**Conclusion:**

The investigated intra-annular SE THV results in excellent 30-day outcomes for aortic ViV procedures for failing surgical bioprostheses or THV. Despite the intra-annular design, hemodynamic results were excellent, even in small bioprostheses. Ease of use of this valve platform is reflected by only two cycles of resheathing in five ViV procedures with hemodynamic stability during all steps of valve deployment.

## Introduction

Valve-in-valve (ViV) transfemoral (TF) transcatheter aortic valve implantation (TAVI) for failing aortic surgical bioprostheses or transcatheter heart valves (THV) has demonstrated a reasonable clinical and hemodynamic efficacy in patients not suitable for redo surgical aortic valve replacement (SAVR) (1, 2). Therefore, this technique was implemented in international guidelines for treatment of valvular heart disease (3, 4). However, certain drawbacks of this procedure were especially described concerning suboptimal hemodynamic results in patients with small bioprostheses or risk of coronary occlusion in patients with low coronary ostia take-off (5, 6). In the past, several procedural steps were introduced to overcome these possible complications including bioprosthetic valve fracture (BVF) to optimize hemodynamic results and intentional leaflet laceration to mitigate the risk of coronary occlusion (7, 8). In terms of postinterventional hemodynamic results, traditionally self-expanding (SE) supra-annular THV are considered to present superior hemodynamics compared with balloon-expandable (BE) intra-annular THV (9, 10). However, apart from a single-case report (11), so far no data are found on the use of a latest-generation SE intra-annular THV for ViV TF-TAVI in failing aortic surgical bioprostheses or THV. The Navitor THV (Abbott, Chicago, IL, USA) consists of a bovine pericardium bioprosthesis mounted in a nitinol frame covering an annulus range from 19 mm to 27 mm. The FlexNav Delivery integrated sheath system presents a True ID of 18 Fr. for the 23 and 25 mm THV and 19 Fr. for the 27 and 29 mm THV and is compatible with 14/15 Fr. sheathes. We herein report our first experience with aortic ViV procedures using this latest-generation intra-annular SE THV.

## Materials and methods

### Patients

Between May 2022 and November 2022, five consecutive patients [4/5 female with mean age of 76.2 years and mean Society of Thoracic Surgeons predicted risk of mortality (STS PROM) score of 2.9%] received ViV TAVI using this SE intra-annular THV for the treatment of failing surgical bioprostheses or THV as determined by transthoracic (TTE) and/or transesophageal echocardiography (TEE). Decision to implant this particular THV as well as application of concomitant procedures such as intentional leaflet laceration or BVF was left to operators’ discretion, and no specific anatomical criteria for using this platform were noted. However, aspects included in decision-making for utilization of BVF consisted of size of index valve and anticipated hemodynamic result, height of coronary ostia take-off, sinotubular junction width, and anticipated risk of sinotubular junction sequestration. In particular, in two patients (patient no. 1 and 3) with a labeled size of the index valve of 21 and 23 mm, BVF was performed to optimize hemodynamic results. In these patients, a wide sinotubular junction and high coronary ostia take-off were seen, and therefore risk for coronary occlusion was mostly absent. Contrarily, in a patient with a labeled size of 23 mm (patient no. 5), no BVF was conducted since a narrow sinotubular junction and low coronary ostia take-off were seen. In this particular patient, BVF would have increased the risk for coronary occlusion.

Allocation of patients to ViV TAVI followed current international recommendations (3) after consensus of the local dedicated heart team. According to the Valve Academic Research Consortium 3 (VARC)-3 criteria, a type of bioprosthetic valve dysfunction was structural valve deterioration in all patients (12) with the echocardiographic correlates of significant stenosis in four out of five patients and severe regurgitation in one patient.

### Diagnostic work-up

The preprocedural diagnostic work-up followed institutional standards and was previously described (13): by routine, all patients received preoperative TTE and TEE for evaluation of cardiac functional status. Furthermore, diagnostic work-up included contrast-enhanced, electrocardiogram-gated multislice computed tomography (MSCT). Datasets were analyzed using the 3mensio Medical Imaging Software (3mensio, Medical Imaging, Bilthoven, Netherlands) for determination of adequate THV size as well as assessment of aortic root anatomy and morphology with an emphasis on the distance of coronary arteries to the aortic annulus and sinus width and assessment of aorto-iliac and peripheral vascular status.

All procedures were performed in a specially equipped hybrid operating suite by a dedicated team of cardiologists, cardiac surgeons, and anesthesiologists. THV function was assessed by invasive measurements of hemodynamics, aortic root angiography, and TTE.

### Study procedure

Institutional standards for aortic ViV procedures were previously described (14). In brief, all herein described procedures were performed via TF access with local anesthesia as first-line approach, except for patients in which intentional leaflet laceration was performed where general anesthesia was used to enable TEE guidance. Utilized vascular closure systems consisted of suture-based devices (ProGlide/ProStyle; Abbott, Chicago, IL, USA) or a collagen plug-based device (MANTA; Teleflex, Wayne, PA, USA). In all patients without intentional leaflet laceration, a single femoral puncture as interventional access was performed, and non-interventional access was conducted via the right-sided radial artery. In one patient (patient no. 4), a single-puncture ViV TAVI without non-interventional access was performed. Target height for THV valve deployment was alignment of both lower stent rims to create optimal postinterventional hemodynamics with full stent deployment of the THV. In one patient with low coronary ostia take-off, intentional deep implantation into a Sapien 3 Ultra (Edwards Lifesciences, Irvine, CA, USA) was performed to protect against coronary occlusion. Intentional leaflet laceration was performed as previously described (15). BVF was performed prior to THV implantation and independently from the anticipated hemodynamic result. Following the procedure, all patients were transferred to a postoperative holding area until the first postoperative day and further stayed until discharge was completed on the ward.

### Statistical analyses

Baseline and intraprocedural data were retrospectively collected, entered into a standardized database, and analyzed. Clinical endpoints were adjudicated in accordance with the updated standardized VARC-3 definitions (12). Patients were presented isolated in tables. For summarized data, absolute numbers were given for categorical variables and mean values for continuous variables.

## Results

### Baseline demographics

Patients presented with a moderate co-morbidity burden (one patient with concomitant coronary artery disease, one patient with s/p stroke, one patient with clinically significant chronic obstructive pulmonary disease) as reflected by the low mean STS PROM score of 2.9%. Three out of five patients were severely symptomatic in New York Heart Association (NYHA) functional class ≥III. Left ventricular function was preserved in all cases. Implanted bioprostheses during index procedures consisted of two surgical bovine pericardial valves [Perimount 21 and 23 mm (Edwards Lifesciences, Irvine, CA, USA)], two surgical porcine valves [Hancock 27 mm and Mosaic 23 mm (Medtronic, Minneapolis, MS, USA)], and one THV (Sapien 3, 26 mm). The range of time interval to ViV procedure was 70–165 months.

Detailed patient demographics are summarized in Table 1.

**Table 1:** Baseline data.

	Patient no.					Mean/Σ
	1	2	3	4	5	
Age range, years	70–75	80–85	75–80	70–75	80–85	76.2
Gender, male/female	Female	Female	Female	Male	Female	4/5 Female
BMI, kg/m^2^	24	19	32	22	22	23.8
STS PROM score, %	2.1	4.1	1.1	1.3	5.8	2.9
Index procedure						
Bioprosthesis type/size, mm	Perimount, 21	Sapien 3, 26	Perimount, 23	Hancock, 27	Mosaic, 23	/
Year of implant	2009	2014	2016	2015	2008	/
Time to valve-in-valve, months	153	92	70	84	165	112
Mode of valve failure^b^	1	1	1	1	1	/
Arterial hypertension, **✓**/✗	**✓**	✗	**✓**	✗	**✓**	3/5 **✓**
Prior Stroke, **✓**/✗	✗	✗	✗	✗	**✓**	1/5 **✓**
Coronary artery disease, **✓**/✗	✗	✗	**✓**	✗	✗	1/5 **✓**
Extracardiac arteriopathy^a^,**✓**/✗	✗	✗	✗	✗	**✓**	1/5 **✓**
Arrhythmia, **✓**/✗	✗	✗	✗	✗	**✓**	1/5 **✓**
COPD^a^ > Gold II, **✓**/✗	✗	**✓**	✗	✗	✗	1/5 **✓**
Creatinine, mg/dl	0.8	0.5	0.7	0.8	0.9	0.7
Pulmonary hypertension^a^>60 mmHg, **✓**/✗	✗	✗	✗	✗	✗	0/5 **✓**
NYHA ≥ III, **✓**/✗	**✓**	**✓**	✗	✗	**✓**	3/5 **✓**
LVEF, %	62	60	62	55	55	58.8

LVEF, left ventricular ejection fraction; BMI, body mass index; COPD, chronic obstructive pulmonary disease.

^a^ Extracardiac arteriopathy and COPD according to EuroSCORE definitions.

^b^ According to VARC-3 definitions (1) Structural valve deterioration, (2) non-structural valve dysfunction (patient–prosthesis mismatch, paravalvular regurgitation, other), (3) thrombosis, and (4) endocarditis.

### Periprocedural data

**Figure 1:**
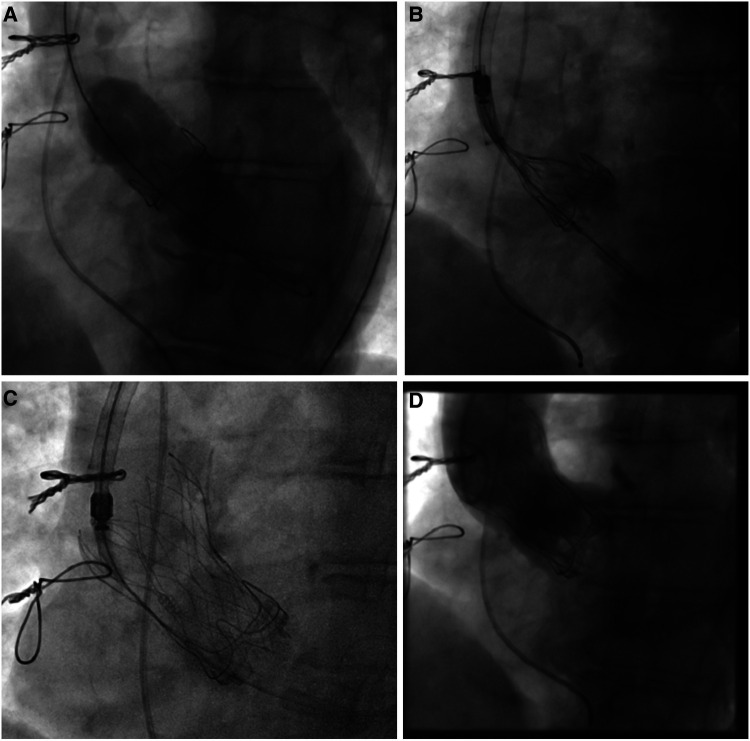
Valve-in-valve procedure using a self-expanding intra-annular transcatheter heart valve for treatment of a failing bovine surgical bioprosthesis. (**A**) Bioprosthetic valve fracture of a 21 mm (True ID: 19 mm) Perimount surgical bioprosthesis using a 22 mm True Dilatation Balloon. (**B**) Placement of a 23 mm self-expanding intra-annular transcatheter heart valve into the surgical bioprosthesis and alignment of both stent inflows. (**C**) Final position with (**D**) absence of any paravalvular leakage.

**Figure 2:**
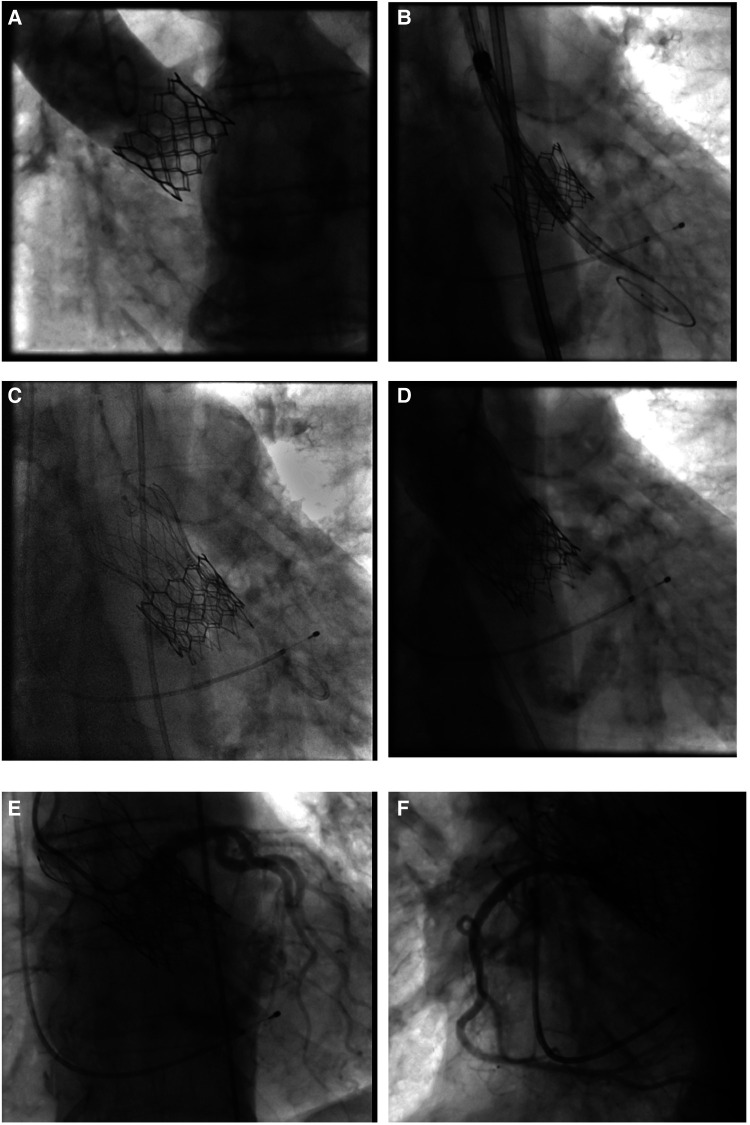
Valve-in-valve procedure using a self-expanding intra-annular transcatheter heart valve for treatment of a failing balloon-expandable transcatheter heart valve. (**A**) Failing balloon-expandable transcatheter heart valve in fluoroscopy with adequate distance of coronary arteries to the aortic annulus and marginal sinus width. (**B**) Placement of a 25 mm self-expanding intra-annular transcatheter heart valve into the Sapien 3, 26 mm (True ID: 21.5 mm) in deep position to protect against coronary occlusion. (**C**) Final position with (**D**) absence of paravalvular leakage. (**E,F**) Sufficient coronary perfusion in selective angiography of coronary arteries.

**Figure 3:**
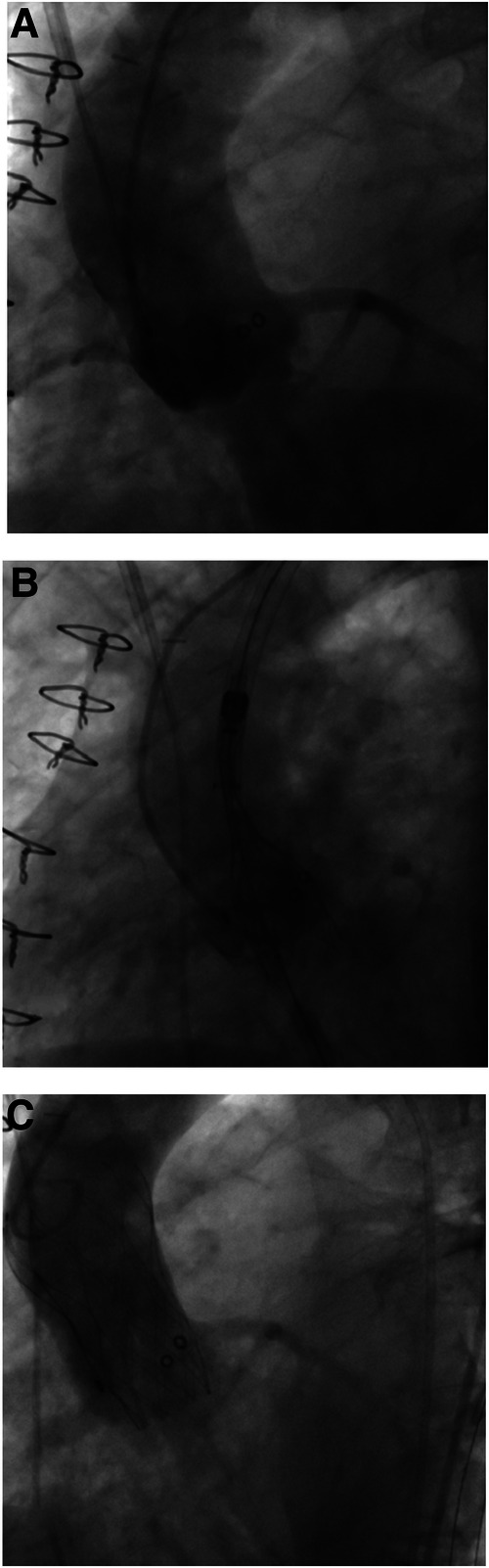
Valve-in-valve procedure using a self-expanding intra-annular transcatheter heart valve for treatment of a failing porcine surgical bioprosthesis. (**A**) Failing 23 mm (True ID: 19 mm) Mosaic surgical bioprosthesis with severe valvular regurgitation. (**B**) Placement of a 23 mm self-expanding intra-annular transcatheter heart valve into the surgical bioprosthesis and alignment of both lower stent parts. (**C**) Final position with absence of any paravalvular leakage.

Mean peak/mean pressure gradients of degenerated bioprostheses with stenosis were 59.8/37.0 mmHg as determined by preprocedural TTE and/or TEE. In one patient, the leading cause for ViV procedure was severe valvular regurgitation. The range of procedure time, fluoroscopy time, and contrast agent used were 14–93 min, 8–28 min, and 50–143 ml, respectively. A cerebral protection system (SENTINEL™ Cerebral Protection System, Boston Scientific Co., Marlborough, MS, USA) was utilized in two patients. Intentional leaflet laceration was performed in one patient and BVF in two patients prior to THV deployment. Balloon pre- and postdilation were performed in one and two patients, respectively. Invasive measurements of mean pre- and post-implant pressure gradients revealed a decrease of peak gradient from 45.2 to 3.8 mmHg and decrease of mean gradient from 51.4 to 13.4 mmHg. Detailed periprocedural data are summarized in Table 2. For an illustration of ViV procedures using this SE intra-annular THV in surgical bovine, porcine valves, and THV, see Figures 1–3.

**Table 2:** Periprocedural data.

	Patient no.					Mean/Σ
	1	2	3	4	5	
Baseline peak gradient, mmHg	50	69	72	48	24	52.6
Baseline mean gradient, mmHg	30	43	47	28	14	32.4
Invasive pre-implant mean gradient, mmHg	65	56	59	49	28	51.4
Aortic regurgitation, grade	0	1	1	2	3	/
Anesthesia, general/CS/LA	LA	LA	General	LA	LA	4/5 LA
Procedure time, min	55	71	93	14	16	49.8
Fluoroscopy time, min	20	27	28	10	8	18.6
Contrast agent, ml	74	143	115	50	80	92.4
True ID index valve (MSCT), mm	19	21.5	21	22	19	/
THV size, mm	23	25	25	25	23	/
Resheathing, n	1	0	0	1	0	2/5 **✓**
Predilatation, **✓**/✗	✗	✗	✗	✗	**✓**	1/5 **✓**
Postdilatation, **✓**/✗	✗	**✓**	✗	✗	**✓**	2/5 **✓**
Cerebral protection, **✓**/✗	**✓**	✗	**✓**	✗	✗	2/5 **✓**
Bioprosthetic valve fracture, **✓**/✗	**✓**	✗	**✓**	✗	✗	2/5 **✓**
Intentional leaflet laceration, **✓**/✗	✗	✗	**✓**	✗	✗	1/5 **✓**
Invasive post-implant mean gradient, mmHg	14	13	15	9	16	13.4

EOA, effective orifice area; CS, conscious sedation; LA, local anesthesia; ID, inner diameter.

### Echocardiographic and clinical outcome data at 30 days

In the study group, peak and mean transvalvular gradients as determined by TTE decreased from 52.6 to 11.6 mmHg and from 32.4 to 6.0 mmHg, respectively. Complete absence of paravalvular leakage (PVL) was documented in all patients.

All-cause 30-day mortality was 0%. Device success, technical success, and early safety were reported to be 100%, respectively. No VARC-3 adjudicated clinical endpoints (disabling stroke, myocardial infarction, acute kidney injury, PPI, access site complication, bleeding) occurred. The range of holding area and hospital stay were 1 and 5–15 days, respectively.

Detailed echocardiographic and clinical outcome data are summarized in Table 3.

**Table 3:** Clinical and echocardiographic results at discharge.

	Patient no.					Σ
	1	2	3	4	5	
All-cause mortality (30 days), **✓**/✗	✗	✗	✗	✗	✗	0/5 **✓**
Cardiovascular or unknown, **✓**/✗	✗	✗	✗	✗	✗	0/5 **✓**
Stroke, **✓**/✗	✗	✗	✗	✗	✗	0/5 **✓**
Myocardial infarction, **✓**/✗	✗	✗	✗	✗	✗	0/5 **✓**
Bleeding (major/life-threatening), **✓**/✗	✗	✗	✗	✗	✗	0/5 **✓**
Access site complications (major), **✓**/✗	✗	✗	✗	✗	✗	0/5 **✓**
Acute kidney injury (AKIN^a^ 2, 3), **✓**/✗	✗	✗	✗	✗	✗	0/5 **✓**
PPM implantation, **✓**/✗	✗	✗	✗	✗	✗	0/5 **✓**
Device success^b^, **✓**/✗	**✓**	**✓**	**✓**	**✓**	**✓**	5/5 **✓**
Early safety^c^, **✓**/✗	**✓**	**✓**	**✓**	**✓**	**✓**	5/5 **✓**
Holding area, days	1	1	1	1	1	1
In-hospital stay, days	5	15	6	5	8	7.8
Peak gradient, mmHg (TTE at 30 days)	11	9	15	19	4	11.6
Mean gradient, mmHg (TTE at 30 days)	7	4	8	9	2	6.0
Any PVL, ✓/✗ (TTE at 30 days)	✗	✗	✗	✗	✗	0/5 **✓**

PPM, permanent pacemaker.

^a^ AKIN, Acute Kidney Injury Network; VARC-3 definitions.

^b^ Device success: technical success + absence of procedural mortality, correct positioning of a single prosthetic heart valve into the proper anatomical position, intended performance of the prosthetic heart valve (no patient–prosthesis mismatch and mean aortic valve gradient of <20 mmHg or peak velocity of <3 m/s and no moderate or severe prosthetic valve regurgitation).

^c^ Early safety at 30 days: all-cause mortality (at 30 days), all stroke (disabling and non-disabling), life-threatening bleeding, acute kidney injury stage 2 or 3 (including renal replacement therapy), coronary artery obstruction requiring intervention, major vascular complication, and valve-related dysfunction requiring repeat procedure (BAV, TAVI, or SAVR).

## Discussion

The main findings of the herein investigated patient series are as follows: (1) this SE intra-annular THV for ViV procedures resulted in excellent acute outcomes with the absence of mortality or VARC-3 adjudicated clinical endpoints; (2) despite the intra-annular design, hemodynamic results were excellent with no PVL and postinterventional single-digit mean transvalvular pressure gradients in all patients even in a bioprosthesis with a diameter of 21 mm; and (3) ease of use of this THV platform in aortic ViV procedures was reflected by only two cycles of resheathing in five ViV procedures with hemodynamic stability during all steps of valve deployment in the herein investigated patients.

Hemodynamic advantages of supra-annular THV for aortic ViV procedures, especially in small aortic bioprostheses with a diameter of ≤21 mm, are well documented by recent prospective randomized data (9). In the VIVID Registry investigating 1,006 patients, postprocedural peak/mean transvalvular pressure gradients of 27.1 ± 13.6/14.7 ± 8.2 mmHg for SE THV and 30.8 ± 15.8/17.7 ± 9.5 mmHg for BE THV (*p* < 0.001 for both values) were reported. Although this may not directly translate into increased mortality in the long term, higher incidences of patient–prosthesis mismatch with consecutive inadequate relief of valve stenosis and symptoms are likely with BE intra-annular THV. Furthermore, an increase of reintervention rates with intra-annular valves, especially in small aortic bioprostheses due to pin-wheeling effects caused by stent underexpansion, and possibility of early valve thrombosis were documented (1). The same is true for TAVI in TAVI procedures. A recent study demonstrated superior device success with SE THV in THV procedures compared with BE THV in THV procedures due to lower postprocedural mean transvalvular gradients with SE devices [SE THV: 10.3 mmHg (8.9–11.7 mmHg) vs. BE THV: 15.2 mmHg (13.2–17.1 mmHg); *p <* 0.001] (16). However, whether superiority of supra-annular SE THV in ViV procedures is an effect of the supra-annular valve design itself or is caused by different design properties of the respective THV still needs to be clarified. Although the herein investigated patient collective is small, postoperative hemodynamics are exceptionally favorable for ViV procedures using an intra-annular THV, even though it has to be emphasized that the herein documented hemodynamic results may be prone to bias since in two patients BVF was performed prior to THV implantation and hemodynamic performance of the utilized THV was determined after BVF. Furthermore, recent data suggest a beneficial impact of BVF after THV implantation with superior long-term effective orifice area (7), while still a patient-specific approach regarding utilization of BVF should be made for every patient to balance the anticipated hemodynamic result against the risk of coronary occlusion. However, these results suggest that not only valve position is crucial for postinterventional hemodynamics in ViV procedures but also the stent design itself. Here, the non-tapered stent and the low nitinol density and large stent cell design of the presented THV may contribute to single-digit mean gradients in all herein investigated cases. This assumption is further underlined by the good hemodynamic results of the predecessor THV platform in small native aortic annuli with severe aortic valve stenosis (17).

This finding may be of special importance for future concepts of lifetime management of aortic valve stenosis. Several concepts were introduced to provide patients with aortic valve stenosis a reasonable treatment strategy when multiple treatments of the aortic valve become necessary, consisting of SAVR-redo SAVR-TAVI or SAVR-TAVI-TAVI sequences (18). Especially in the latter concept, an initial ViV procedure using an intra-annular BE THV might be advantageous to facilitate a possible second ViV procedure using SE THV. In this scenario, a possibly unfavorable hemodynamic result with early THV failure after the first ViV treatment is accepted to enable a second ViV procedure. With the herein utilized intra-annular valve system, it seems possible to combine excellent acute hemodynamic results and anticipated extended valve durability of SE THV with preservation of valve reintervention, i.e., a second ViV procedure. Nevertheless, these preliminary results have to be confirmed in larger patient cohorts, and proof of concept of placing a supra-annular SE THV in an intra-annular SE THV is pending.

## Limitations

Limitations are inherent in the retrospective, single-center study design with limited patient numbers: patients were not randomized to a specific treatment or valve; therefore, patient preselection with hidden confounders may apply.

## Conclusion

The investigated SE intra-annular THV resulted in reasonable 30-day outcomes in aortic ViV procedures for failing surgical bioprostheses or THV in this small series. Despite the intra-annular design, hemodynamic results were good in the herein investigated failing bioprostheses. Ease of use of this valve platform is reflected by only two cycles of resheathing in five ViV procedures with hemodynamic stability during all steps of valve deployment. A further advantage of using this particular THV in aortic ViV procedures may be the possibility for a second ViV procedure using a supra-annular THV, which is a concept that has to be validated in the future.

## Data Availability

The raw data supporting the conclusions of this article will be made available by the authors, without undue reservation.
